# A questionnaire for assessing breastfeeding intentions and practices in Nigeria: validity, reliability and translation

**DOI:** 10.1186/s12884-017-1366-9

**Published:** 2017-06-07

**Authors:** Andy Emmanuel, Sheila E. Clow

**Affiliations:** Division of Nursing and Midwifery, Department of Health and Rehabilitation Sciences, University of Cape Town, Cape Town, South Africa

**Keywords:** Breastfeeding practices, Breastfeeding intention, Validity and reliability, Infant feeding practices, Infant feeding intention, Hausa, Irish national infant feeding survey questionnaire

## Abstract

**Background:**

Validating a questionnaire/instrument (whether developed or adapted) before proceeding to the field for data collection is important. This article presents the modification of an Irish questionnaire for a Nigerian setting. The validation process and reliability testing of this questionnaire (which was used in assessing previous breastfeeding practices and breastfeeding intentions of pregnant women in English and Hausa languages) were also presented.

**Method:**

Five experts in the field of breastfeeding and infant feeding voluntarily and independently evaluated the instrument. The experts evaluated the various items of the questionnaire based on relevance, clarity, simplicity and ambiguity on a Likert scale of 4. The analysis was performed to determine the content validity index (CVI).Two language experts performed the translation and back-translation. Ten pregnant women completed questionnaires which were evaluated for internal consistency. Two other pregnant women completed the questionnaire twice at an interval of two weeks to test the reliability. SPSS version 21 was used to calculate the coefficient of reliability.

**Results:**

The content validity index was high (0.94 for relevance, clarity and ambiguity and 0.96 for simplicity). The analysis suggested that four of the seventy one items should be removed.

Cronbach’s Alpha was 0.81, while the reliability coefficient was 0.76. The emerged validated questionnaire was translated from English to Hausa, then, back-translated into English and compared for accuracy.

**Conclusion:**

The final instrument is reliable and valid for data collection on breastfeeding in Nigeria among English and Hausa speakers. Therefore, the instrument is recommended for use in assessing breastfeeding intention and practices in Nigeria.

**Electronic supplementary material:**

The online version of this article (doi:10.1186/s12884-017-1366-9) contains supplementary material, which is available to authorized users.

## Background

In sub-Saharan Africa, at least 1.16 million newborns die each year [1]. Breastfeeding is one of the immediate newborn care interventions that reasonably reduces neonatal mortality [2]. Exclusive breastfeeding for the first six months of infant life has been shown to improve child survival [3] and can save 140,000 newborns every year in Africa [1]. Although breastfeeding is universal in sub-Saharan Africa, only 33% of women breastfeed exclusively for six months [1]. The exclusive breastfeeding rate is even lower in Nigeria where it is estimated at 17% [4]. Nigeria has the highest infant and neonatal mortality rates in the world (128/1000 and 37/1000 respectively) [4, 5]. The inverse relationship between infant mortality and the rate of exclusive breastfeeding suggests that increasing the rate of exclusive breastfeeding could reduce infant mortality in Nigeria.

Currently there is no data indicating what factors affect the uptake of breastfeeding in Nigeria. Thus there is the need for the development of a valid and reliable instrument that is sensitive to local context and available in a local language. This could be used to establish current infant feeding practices and to inform policy makers and practitioners on the areas that require specific interventions to improve infant feeding policies and practices nationally. This article presents a questionnaire that was modified from the Irish national infant feeding survey [6]. The instrument was validated and translated for use in Nigeria for assessing factors that influence infant feeding intentions and practices.

Validity ensures that an instrument measures what it is designed to measure. No instrument is completely valid, but it is possible to determine the degree of validity, rather than only ascertaining if validity exists [7, 8]. Since validity may vary from sample to sample and from one situation to another, it means that validity testing must evaluate the use of an instrument for a specific group or purpose [7]. This realisation informed the validation process for the instrument that was adapted.

Of the over 35 terms used to describe the different kinds of validity [8], only content, criterion-related and construct validities are commonly used [9]. Content validity and the reliability coefficient thus guided the process of validation of this instrument because these criteria were able to establish a statistical value of the logic relationship between items of an instrument and its purpose [8] and because the instrument was not a newly developed one. Lynn [8] posited that an instrument is developed in two stages. They are development and judgement/quantification stages.

The development stage involves the identification of the content domain by conducting an extensive literature review. In this case, for example, because an instrument was being developed to assess the determinants of exclusive breastfeeding, all determinants needed to be identified and categorized. The next step of the development stage involves sampling and generating relevant items from the content domain and assimilating and sorting these items into a useable form.

The judgement/quantification stage involves the quantification of the content validity (CV) of items, followed by the judgement of the CVI of the instrument [8]. After successfully developing and validating an instrument, it is necessary to test it for reliability and consider translating it to the local languages of target population.

Prior to this study no data existed on testing of instruments for assessing infant feeding in a Nigerian setting. It is possible that researchers in the area of infant feeding adopted instruments from studies conducted in other countries without contextualizing them.

The objectives of this study were to identify and validate an instrument for assessing infant feeding intention and practices in a Nigerian setting, to test this instrument for reliability and to translate it into a local Nigerian language.

## Method

### Design

This study adopted descriptive design. The study is a product of a study carried out in Plateau State, north-central Nigeria to determine breastfeeding intentions and practice of women. It was conducted among pregnant women who have had a child within the last three years.

### Search for instrument

Instruments or tools used in previous studies were searched in Medline through PUBMED, Google scholar, CINAHL and EBSCO using key words like breastfeeding or infant feeding or mixed feeding and questionnaire or instrument. Articles published in English from 2005 to date were included. The search returned several articles in the first instance. After an assessment of the articles, five authors were contacted through email and telephone to provide copies of their instruments for possible adoption. Only one study from Nigeria replied. The available instrument was evaluated but found inappropriate for the intended study because the Nigerian instrument was developed to collect data about current breastfeeding practices among female medical doctors. A general Google search was performed and an Ireland study was retrieved. It contains items that would be appropriate for the intended study. This instrument was considered for use because it was the most current study we found that has a tool that can be modified for the intended study. The original tool was adapted from Bolling et al. [10] and validated and tested for reliability before use in Ireland.

### Adaption/development of instrument

The aims of the Ireland study were to describe infant feeding, determine the rate and duration of breastfeeding and the factors that influenced women in Ireland to breastfeed [6]. The Irish study was administered in three phases. The first phase was between birth and 48 h, the second phase was between three and four months after birth; and the final phase was between six and seven months after birth. Women answered an average of 45 questions per phase (135 questions in total). The Irish instrument used in the Irish study was developed and validated by the researchers. A reliability test was also performed prior to use for the study. According to the objectives of the current study and guided by the constructs of the Health Belief model, 78 items were drawn from the Irish questionnaire. After a two-phase departmental review process, a total of 71 items were included.

Changes were made to the tense of questions because the Irish survey was prospective. However some of the items of the new questionnaire will also retrieve information prospectively. Changes were also made to the wording of some of the options of questions asked in order to suit the Nigerian setting. For example, “lactation consultant” is not a familiar term in this setting; some of these were substituted for familiar terms. Where options like nurse and midwife were presented separately in the original questionnaire, they were merged because participants would not understand the difference between a midwife and a nurse. In Nigeria, nurses who work in maternity unit are not distinguished from midwives because they carry out the same duties and wear the same uniform. As such, the target population regards them as the same. Some closed questions were changed to open-ended questions to enable respondents express themselves fully.

Lynn [8] recommended that three to ten experts should be consulted in the judgment and quantification stage of determining the validity of the instrument’s content. He further stated that three experts may be used where accessible and agreeable experts are difficult to locate, and ten where they are available. Depending on the number of judges, there are different rules for the level of agreement required. In this case, five judges were regarded as sufficient for the quantification phase, because the instrument was not newly developed but adapted from a similar study. Participants were selected using purposive sampling. The selected judges are familiar with the sociocultural practices in the State and had conducted related studies. Three of the judges were registered nurses from the University Teaching Hospital in Jos were trained in the Baby Friendly Hospital Initiative. The remaining two were lecturers in the Department of Nursing Science, University of Jos; one of these was a midwifery lecturer, while the other was a medical sociologist who had conducted a study on the social dimensions of exclusive breastfeeding and its impact on child survival and development [11].

### Judgment and quantification stage

Content Validity Index (CVI) and the coefficient of reliability guided the validation of the instrument. Lynn [8] focused on relevance as the only determinant of content validity, but Yaghmale [12] added 3 other determinants, namely, simplicity, clarity and ambiguity. The instrument for data collection was presented to the panel of five judges, instructing them to assess its relevance, clarity, simplicity and ambiguity on an ordinal Likert scale of four. The judges were provided with the scoring system as follows:1 = not relevant, 2 = item needs some revision, 3 = relevant but needs minor revision and 4 = very relevant (please see Table 1). The same scoring applied to clarity, simplicity and ambiguity [12]. The judges were also given a guide on how to carry out the assessment using the information presented in Table 1 as well as the aim and objectives of the study.

**Table 1:** Criteria for measuring content validity [12] for each item on the questionnaire

SCORES/CHARACTERISTICS	1	2	3	4
Relevance	Not relevant	Item needs some revision	Relevant but needs minor revision	Very relevant
Clarity	Not clear	Item needs some revision	Clear but needs minor revision	Very clear
Simplicity	Not simple	Item needs some revision	Simple but needs minor revision	Very simple
Ambiguity	Doubtful	Item needs some revision	No doubt but needs minor revision	Meaning is clear

Proportions were used to calculate CVI for each item and the whole instrument. The CVI of each item was determined as a proportion of judges who had judged an item valid (i.e. obtaining a score of 3 or 4).The proportion of judges who had scored an item 3 or 4 on each parameter (relevance simplicity, clarity, ambiguity) was first determined. CVI of each item = the proportion of experts who rated the item content valid (score of 3 or 4) [9, 13]. In other words, it refers to the number of judges who scored an item as valid, divided by the total number of judges.

The content validity of the instrument with respect to the four parameters was then determined as a proportion of items which had a CVI of 0.75 to 1.00. For the instrument to be content valid, items should score 3 or 4 on a Likert scale of 4 [9]. Therefore the items that had a CVI of over 0.75 would remain in the questionnaire.

### Internal consistency and reliability

Ten women participated in the internal consistency test. They voluntarily completed the questionnaires and analysis was performed by a statistician to determine the internal consistency. Two other women participated voluntarily in the reliability test. Altogether, 12 women were conveniently selected for the internal consistency and reliability tests and this sample was determined by a statistician. They were recruited from Plateau state, northern Nigeria.

The inclusion criterion was that a woman could participate in the study if she had had at least a child previously and is pregnant with another. This is because the instrument elicited information regarding previous breastfeeding practices and at the same time breastfeeding intention. Women whose last children did not live at least 24 h were excluded because they may not have information for previous breastfeeding history. Women attending antenatal care who made the inclusion criteria were invited to participate.

Reliability was analysed using the reliability coefficient, and analysis of internal consistency used Cronbach’s alpha. The analysis was done by a statistician using SPSS version 20. A pilot study was carried out to further test the instrument. This is being processed for publication elsewhere.

### Translation

Two people who had received formal training in both English and Hausa languages were recruited for translation. The translation from English to Hausa was performed by a trained language expert at tertiary level. While the reverse translation (from Hausa to English) was performed by a different person who is a medical personnel with Hausa as his first language and had also received formal language training at secondary school level.

Hausa is the language that is commonly used for communication in this setting. The researcher anticipated that most of the respondents might not understand English to the level required to provide the information needed. Therefore, it was necessary to translate the validated instrument into Hausa. This was done as follows:

The validated instrument (Questionnaire A) was given to a person who was fluent in both English and Hausa to translate the English instrument into Hausa. Thereafter, the Hausa version (Questionnaire B) was given to another person who was also fluent in both languages to translate it back into English. The new English version (Questionnaire C) was compared with Questionnaire A.

### Ethical consideration

Ethical approval was obtained from the Human Research Ethics Committee of the Faculty of Health Sciences, University of Cape Town (HREC REF: 316/2014). Ethical approval was also obtained from Plateau State to conduct this study. Furthermore, permission was obtained from the Plateau State Ministry of Health and Plateau Specialist Hospital to conduct this study. Informed consent was obtained from all participants in writing. All women voluntarily participated in the study and were assured of anonymity and confidentiality.

## Results

The results for content validity, reliability, internal consistency and translation are presented.

### Content validity

The CVI of all items with respect to relevance, clarity, simplicity, and ambiguity was calculated. Sixty-seven (67) of the seventy-one (71) items were judged valid for relavance, clarity, and ambiguity respectively. Therefore, CVI = 67/71 = 0.94 meaning that 94% of items had a CVI of ≥0.75 for these parameters. Sixty-eight of the seventy-one items were judged valid for simplicity. CVI (Simplicity) = 68/71 = 0.96 and implies that 96% of items were judged valid for simplicity.CVI of the entire instrument was calculated after determining the CVI of each of the items.

The CVI of the questionnaire is the proportion of total items rated as valid (i.e. CVI of 0.75 to 1.00) [9]. The CVI of this instrument is 0.94.

Four items (items 9, 25, 34 and 70) were withdrawn for not reaching this threshold. (please see Table 2 for scores by judges) Item 9 was a question about the perception of participants about their economic status; item 23 was about antenatal attendance in the previous pregnancy, item 34 was if someone had ever advice participants to breastfeed or stop breastfeeding or take a prescribes drug, while item 70 was about commencing work within two years after birth.

**Table 2:** Content validity index of each item for relevance, clarity, simplicity and ambiguity

ITEM	RELEVANCE	CLARITY	SIMPLICITY	AMBIGUITY
1	0.80	1.00	1.00	1.00
2	1.00	1.00	1.00	1.00
3	0.80	0.80	0.80	1.00
4	1.00	1.00	1.00	1.00
5	1.00	1.00	1.00	1.00
6	1.00	1.00	1.00	1.00
7	1.00	1.00	1.00	1.00
8	0.80	1.00	1.00	0.80
9	0.60	0.80	0.80	0.80
10	1.00	1.00	1.00	1.00
11	1.00	0.80	0.80	0.80
12	1.00	0.80	1.00	0.80
13	1.00	1.00	1.00	1.00
14	1.00	1.00	1.00	1.00
15	1.00	0.80	0.80	0.80
16	1.00	0.80	0.80	0.80
17	1.00	1.00	0.80	0.80
18	1.00	1.00	1.00	1.00
19	1.00	0.60	0.60	0.40
20	1.00	1.00	1.00	1.00
21	1.00	1.00	1.00	1.00
22	1.00	1.00	1.00	1.00
23	1.00	1.00	1.00	1.00
24	1.00	1.00	1.00	1.00
25	0.60	0.80	0.80	0.80
26	0.80	0.80	0.80	0.80
27	1.00	1.00	1.00	1.00
28	1.00	1.00	1.00	1.00
29	0.80	0.80	0.80	0.80
30	1.00	1.00	1.00	1.00
31	1.00	0.80	1.00	0.80
32	0.80	1.00	1.00	1.00
33	0.80	0.80	0.80	0.80
34	0.60	0.80	0.80	0.80
35	0.80	0.80	0.80	0.80
36	0.80	0.80	0.80	0.80
37	1.00	1.00	1.00	1.00
38	0.80	0.80	0.80	0.80
39	0.80	1.00	1.00	1.00
40	0.80	1.00	1.00	1.00
41	0.80	1.00	1.00	1.00
42	1.00	1.00	1.00	1.00
43	1.00	0.80	0.80	0.80
44	1.00	1.00	1.00	1.00
45	1.00	0.60	0.80	0.60
46	1.00	1.00	1.00	1.00
47	1.00	0.80	1.00	0.80
48	1.00	1.00	1.00	1.00
49	1.00	1.00	0.80	0.80
50	1.00	0.80	1.00	0.80
51	0.80	1.00	1.00	1.00
52	1.00	0.80	0.80	0.80
53	0.80	0.60	0.60	0.60
54	1.00	1.00	0.80	1.00
55	0.80	0.80	0.80	0.80
56	1.00	1.00	1.00	1.00
57	1.00	1.00	1.00	1.00
58	1.00	1.00	1.00	0.80
59	1.00	1.00	1.00	0.80
60	1.00	1.00	1.00	1.00
61	1.00	1.00	1.00	1.00
62	0.80	0.80	0.80	0.80
63	1.00	1.00	1.00	1.00
64	1.00	1.00	1.00	1.00
65	1.00	1.00	1.00	1.00
66	1.00	1.00	1.00	1.00
67	1.00	0.80	1.00	1.00
68	1.00	0.80	1.00	1.00
69	0.80	0.80	0.80	0.80
70	0.60	0.40	0.40	0.40
71	1.00	0.80	0.80	0.80

### Internal consistency and reliability

All women recruited for the internal consistency and reliability study had formal education and could read and write in English. The average age of the women was 28.4 years (standards deviation was 2.3). All women for this study were recruited from Jos North local government area, Plateau state Nigeria.

Cronbach’s Alpha measuring internal consistency was calculated to be 0.81, while the reliability coefficient was 0.76.These results suggest that the instrument is internally consistent and reliable for this population.

### Translation

The comparison revealed that some of the items in Questionnaire B needed to be revised. A vacuum extractor was translated to mean “a machine that assists delivery”. This is because there is no Hausa word for “vacuum extractor”. General anaesthesia was inaccurately translated as analgesia. This was corrected to “*maganin sa barci”*, meaning “anaesthesia”. “At what time intervals do you feed your baby” was corrected to read “time of commencement of feeding”. The phrase “baby not matured enough to feed” was corrected to read “baby was not feeding”. Consequently, a new instrument emerged (Questionnaire D), which was the corrected version of Questionnaire B.

## Discussion

The content validity, reliability and internal consistency of the instrument and translation are discussed below.

### Content validity

The CVI for relevance, clarity and ambiguity (0.94 each) of all the items assessed was high, indicating that 94% of items were judged valid, relevant, clear and unambiguous by the experts. The CVI for simplicity (0.96) was higher than were the values for the other three components. Four items with low scores were removed from the instrument. Three of these four items were assessed below the required threshold for relevance, doubt and clarity. However, one of the four items attend the threshold for simplicity. Lynn [8]advised that items with minimum agreement of experts may be eliminated or revised. If there are many such items, the instrument may need to be re-evaluated by the same experts after it has been revised, so as to obtain sufficient content validity. However, Yaghmale [11] posited that a CVI of 0.80 or higher was accepted and considered sufficient. Since the CVI for all the components was more than 0.80, the instrument was considered sufficient, and the items that received minimum agreement in terms of their relevance were removed because they will elicit similar responses with other items in the instrument. Items 19, 45 and 53 reached the required level of CVI in terms of their relevance but had CVI less than 0.75 for clarity, simplicity and ambiguity. The judges did not make suggestions of rewording in order to address the problems identified with clarity, simplicity and ambiguity. Therefore, the three items were maintained for a possible change after pilot-testing the instrument. Items were checked for clarity, ambiguity and simplicity during the pilot study. The test-retest result indicated a high coefficient of reliability.

### Internal consistency and reliability test

Reliability is concerned with how consistently, the measurement technique is able to measure a variable or a concept, that is, it measures the repeatability of the instrument [7, 9]. The coefficient of reliability was high, suggesting that the instrument is reliable for data collection in this population. Cronbach’s alpha reflects the internal consistency within an instrument and measures how well a set of items is able to measure a particular behaviour or characteristic. Furthermore, Cronbach’s alpha indicates that the instrument is internally consistent. The validated tool is available at the end of the article. However, researcher in Nigeria that intend to use this instrument should consider conducting a reliability test with a larger sample before use.

### Translation

The translation effectively developed a Hausa version of the questionnaire. Questionnaires A and D are suitable to collect data about infant feeding practices in English and Hausa respectively. The two questionnaires are available as Additional files 1 and 2. This will make data collection in Northern Nigeria easier and ensure the collection of accurate data.

### Implication of the instrument

Northern Nigeria has the highest infant mortality in the country [3]. Adapting, validating and translating an infant feeding questionnaire for use among Hausa speaking people in northern Nigeria will be helpful for research on infant feeding. This questionnaire will contribute to understanding the factors that influence infant feeding practices in Nigeria. This may inform the promotion of breastfeeding which in turn has the potential to reduce infant mortality. Therefore, this instrument will contribute to protecting, promoting and supporting breastfeeding and by extension, promoting infant health.

## Conclusion

The modified, validated and translated questionnaire will provide future researchers in Nigeria with a tool for collecting information about infant feeding practices and feeding intention. This article is the first to report the validation of a questionnaire for assessing infant feeding practices in a Nigerian context, and translating it into Hausa.

## Additional files

Additional file 1: Questionnaire A. English version of questionnaire. Original English version of validated questionnaire. (DOCX 22 kb)

Additional file 2: Questionnaire D. Hausa version of questionnaire. The Hausa version of questionnaire translated from questionnaire A. (DOCX 26 kb)
