# Supplementary material for: A questionnaire for assessing breastfeeding intentions and practices in Nigeria: validity, reliability and translation
Source: BMC Pregnancy Childbirth. 2017 Jun 7;17:174. doi: 10.1186/s12884-017-1366-9 (PMC5463374; doi:10.1186/s12884-017-1366-9)
Supplement: Supplementary file 1 — Questionnaire A. English version of questionnaire. Original English version of validated questionnaire. (DOCX 22 kb) [file 12884_2017_1366_MOESM1_ESM.docx]

**Questionnaire on breastfeeding intension and practices of women in plateau state, Nigeria.**

**INSTRUCTIONS**

- If you had twins or multiple births, please answer these questions for the baby who was born first.
- Sometime you are asked to write in a number, please enter number as figure rather than words.
- Sometimes you will be asked to write the answer in your own words.
- For questions with options, tick (✓) or circle the option that best applies to you.(Please pick only one from the options)
- Please be honest with all your answers.

**Section A**: Background information.

1. “Age ……………….........
2. How far is your pregnancy? (in weeks)...................
3. In your previous pregnancy, did you have a single child, twins (or more)?
4. Single birth
5. Twins
6. More than two
7. What is your highest level of educational?
8. No formal education
9. Primary
10. Secondary
11. Tertiary
12. Marital status?
13. Single
14. Married
15. How many children do you have?.......................
16. What is your occupation?.......................................
17. What is the family average monthly earnings?
18. Less than N20,000
19. N20, 000 to N40,000
20. N41,000 to N 80,000
21. N81,000 to N120,000
22. More than N120, 000

**Section B**: breastfeeding practice and related factors.

1. Thinking about the birth itself, what type of delivery did you have?
2. Normal
3. Forceps
4. Vacuum extraction
5. Caesarean section
6. When you were in labour, what kind of pain relief did you have, if any?
7. Epidural or spinal injection
8. Pethidine
9. Gas or air to breathe
10. A general anaesthesia
11. Water
12. Nothing at all
13. Other s (please specify).....................................................................
14. How much did your baby weigh when he/she was born. Please give your answer in Kilograms…………………………………………………………………………
15. Before your last baby was born, how did you plan to feed him/her in the first six months?
16. Breast milk
17. Formula
18. Combination of breast and formula
19. I didn’t have any plans.
20. Why did you think you would feed your baby this way? Please write all reasons……………..

……………………………………………………………………………………………

……………………………………………………………………………………………

……………………………………………………………………………………………

……………………………………………………………………………………………

1. How old was your baby at his or her first feed?
2. Less than 30 minutes
3. Between 30 minutes and 1 hour
4. Between 1 hour and 4 hours
5. More than four hours after delivery.
6. What kind of food did your baby have for his/her first feed?
7. Breast milk.
8. Formula
9. Others (specify)…………………………………………………………………………………………
10. How old was your baby when you first introduced anything other than breast milk........................
11. What was the main reason that led you to choose the way you fed this baby?....................

………………………………………………………………………………………………

………………………………………………………………………………………………………

1. If your baby had any fluid (water and formula) other than breast milk in the early days of breastfeeding, was it because you were advised to or because you wanted your baby to have it.
2. I was advised to give anything else
3. I wanted to give my baby something else
4. I only gave my baby breast milk in the early days.
5. Were there any problems breastfeeding your baby in the early days?.......................

…... if so, what were they……………………………………………………………………

………………………………………………………………………………………

………………………………………………………………………………………

……………………………………………………………………………………………

1. Which of the following best describes your baby’s feeding at one(1) week, six (6) weeks, twelve (12) weeks and twenty four (24) weeks. (Please place a tick in the relevant box for each column. There should only be 1 tick per column and 4 ticks altogether).

|  | 1 week | 6 weeks | 12 weeks | 24 weeks |
| --- | --- | --- | --- | --- |
| Breast milk only |  |  |  |  |
| Formula only |  |  |  |  |
| Breast milk and formula or other foods |  |  |  |  |

1. What are the reasons for feeding your baby this way?

Week one reason (s).................................................................................................................

..............................................................................................................................................

Week six reason (s)………………………………………………………………………………

...................................................................................................................................................

Week twelve reason (s) ………………………………………………………………………………………

..............................................................................................................................................

Week twenty four....................................................................................................................

1. Did you attend any antenatal care when you were pregnant with your previous child?..............
2. Did anyone discussed feeding your baby with you during pregnancy……………………
3. While you were pregnant with your previous baby, did you receive any information about the health benefits of breastfeeding?............................................................
4. Where did you receive this information?.................................................................
5. How have your friends and family fed their children when they were babies?
6. Most of them gave formula
7. Most of them breastfed
8. About half of them formula fed and half breast fed.
9. I don’t know
10. How were you fed when you were a newborn baby?
11. Breastfed
12. Formula fed
13. Breast and formula milk
14. I don’t know
15. If you had previous children, how did you feed them in the first six months?

|  | Breast milk only | Formula only | Combination of formula and breast milk |
| --- | --- | --- | --- |
| Eldest child |  |  |  |
| Second eldest child |  |  |  |
| Third eldest child |  |  |  |
| Fourth eldest child |  |  |  |

1. Was your baby born in the hospital or at home?
2. Hospital
3. Home
4. On the day that you left hospital or at 48 hours if you had a home birth, what most accurately describe how you fed your baby?
5. Breast milk
6. Formula
7. Combination of breast and formula
8. Expressing breast milk
9. My baby was not feeding
10. I don’t remember.
11. Since your baby was born, have you had any of the following as a result of breastfeeding?
12. Mastitis (inflammation of the breast tissue)
13. Thrush
14. Nipple pain
15. None of the above
16. Others specify…………………….
17. Who or what helped you most to continue breastfeeding?
18. Own experience
19. Friends/ other mothers
20. Mother in law
21. Other relatives
22. Health professional (Nurses/Doctors)
23. Peer or support groups
24. Voluntary organizations
25. Books/ magazine/TV
26. Others (please specify)…………………………………………………
27. Who or what helped you least to continue breastfeeding?
28. Own experience
29. Friends/ other mothers
30. Mother in law
31. Other relatives
32. Health professional (Nurses/Doctors)
33. Peer or support groups
34. Voluntary organizations
35. Books/ magazine/TV
36. Others (please specify)…………………………………………………………………………………
37. Who or what influenced you to stop breastfeeding?
38. Own experience
39. Friends/ other mothers
40. Mother in law
41. Other relatives
42. Health professional (Nurses/Doctors)
43. Peer or support groups
44. Voluntary organizations
45. Books/ magazine/TV
46. Others (please specify)…………………………………………………………………………………………
47. Did you have skin to skin contact with your baby after he/she was born (in the first hour after they were born)?
48. Yes
49. No
50. Can’t remember
51. If yes, who assisted you?
52. I was not shown
53. Nurse
54. Nursing/medical student
55. Friends /relative
56. Doctor
57. Ward attendants
58. Others (please specify)………………………………………………………………………………………
59. Did they stay with you while you were breastfeeding?
60. I was not shown
61. Stayed the whole time until the baby was asleep
62. Left once the baby was feeding but came back to check on you
63. Left once the baby was feeding and did not come back to check on you
64. Left before the baby had started feeding.
65. How useful did you find this help?
66. I was not given any help at all at this time
67. Extremely useful
68. Very useful
69. Not very useful
70. Not useful at all.
71. Were there problems breastfeeding your baby in the early days?
72. Yes
73. No

if yes what were they…………………………………………………………………….

……………………………………………………………………………………

……………………………………………………………………………………

……………………………………………………………………………………

1. Did anyone give you help with problem (difficulty with breastfeeding) in the early days?
2. I didn’t have any problem
3. Nurse helped me
4. Doctor helped me
5. Friend/ relative helped me.
6. Members of local support group helped me.
7. Others (please specify)………………………………………………………
8. After you left the hospital, did you receive any home visit from any of the following?
9. Nurse
10. Doctor
11. No visit in the first two weeks
12. Others (please specify)……………………………………………………………………….

If you receive visit, how many did you receive…………………………………..

1. Were you given information about any of the following to help with breastfeeding after you went home?
2. I was not given any information about support services
3. Community breastfeeding support group
4. Others (please specify)………………………………………………………………………………………
5. How easy was it for you to find breastfeeding support services?
6. I did not seek any support services
7. Easy
8. Difficult
9. I was unable to access support services.
10. How do you intend to feed the baby you are pregnant with?
11. Breast milk
12. Formula
13. Combination of breast and formula
14. Why will you feed your baby this way?................................................................................

……………………………………………………………………………………………

…………………………………………………………………………………………

………………………………………………………………………………………………………………………………………….........................................................................................................

1. How long do you intend to feed this baby with breast milk only………………………………
2. Why…………………………………………………………………………………………….....

………………………………………………………………………………………………………

1. At what age of your baby will you stop breastfeeding entirely?…………………………………
2. If you are aware of the benefits of breast feeding for the baby, please write them below…………

………………………………………………………………………………………………………

………………………………………………………………………………………………………

……………………………………………………………………………………………………

1. If you are aware of the benefits of formula feeding for the baby, please write them below

……………………………………………………………………………………

…………………………………………………………………………………………………………………………………………………….........................................................

1. Have you ever seen an advertisement on television, radio or in a magazine or elsewhere for breastfeeding?.......................................................................
2. Have you ever seen an advertisement on television, radio or in a magazine or elsewhere for infant formula?

………………………………………………………………………………………………………………………………………………….........................................................................................................................

1. Thinking about the most helpful information you received about breast feeding since your baby was born, who or what had the most impact on you?
2. Own experience
3. Friends/other mothers
4. Partner
5. Your mother
6. Mother-in-law
7. Sisters
8. Other relatives
9. Health professionals
10. Books and magazines
11. Others (please specify)………………………………………………………………………………………
12. Since your baby was born, have you ever feed him/her in a public place
13. No-I never fed in a public place.
14. Yes-Breastfed in a public place
15. Yes-bottle fed infant formula in a public place
16. Yes – bottle fed expressed breast milk.
17. Have you ever had problems finding somewhere to breastfeed your baby in a public place?.................
18. Have you ever been stopped or made to feel uncomfortable about breastfeeding in a public place?..............
19. Which of the following best describes breastfeeding your baby?
20. I would like to have breastfeed for longer
21. I breastfed for as long as I intended
22. I had breastfed for longer than I had intended
23. For how long (in months or years) did you breast feed your previous baby?.....................
24. If you planned to and started breastfeeding, what were your reasons for stopping breastfeeding?...................................................................................................................

…………………………………………………………………………………

…………………………………………………………………………………………

……………………………………………………………………………………………

SECTION C EMPLOYEMENT.

1. What is the occupation of the baby’s father?................................................................
2. Were you working when you had your previous baby?...................................................
3. How many hours were you working in a day when you had your previous child?...........
4. How old was your baby when you returned to work?..............................................
5. What arrangements (if any) did you make regarding the care of your baby when you are at work?................................................................................................................................

…………………………………………………………………………………………

………………………………………………………………………………………..........

……………………………………………………………………………………........

1. How would you describe your main employment status just before you had your baby?
2. Working for payment or profit
3. Looking for first Job
4. Unemployed
5. Student or pupil
6. Looking after home/family
7. Unable to work due to permanent sickness/disability.
8. Others (please specify)…………………………………………………
9. Did (do) you work as an employee or are (were) you self-employed in your main Job?
10. Employee
11. Self employed, with paid employee
12. Self employed, without paid employee
13. Assisting relative/ others (not receiving a fixed wage or salary)
14. Looking after home / family.
15. Does your employer provide facilities at work for you to express milk or breastfeed your baby if you want to?
16. Yes-to express milk
17. Yes to breastfeed
18. No- neither
19. Not applicable”
