# Supplementary material for: A questionnaire for assessing breastfeeding intentions and practices in Nigeria: validity, reliability and translation
Source: BMC Pregnancy Childbirth. 2017 Jun 7;17:174. doi: 10.1186/s12884-017-1366-9 (PMC5463374; doi:10.1186/s12884-017-1366-9)
Supplement: Supplementary file 2 — Questionnaire D. Hausa version of questionnaire. The Hausa version of questionnaire translated from questionnaire A. (DOCX 26 kb) [file 12884_2017_1366_MOESM2_ESM.docx]

Questionnaire D

**TAMBAYOYI DON SAMUN RA’AYOYIN MATA GAME DA YANDA ZA SU SHAYAS DA YARAN DA ZA SU HAIFA DA MADARAN MAMA (NONO) DA KUMA YAN DA SUKA SHAYAS A BAYA A JIHAR PLATO NA NIJERIYA**

**KA’IDODI**

- In kin taba haifan yanbiyu ko fiye da haka, ki ansa wadannan tambayoyin a madadin yaron da aka haifa na farko
- A wasu wurare za’a bukaci ki rubuta lambobi, sai a rubuta lambobin ba kalmomi ba
- A wasu wurare kuma za’a bukaci ki yi rubutu a kalmomin ki
- A tambayoyin da ke da daman zabe daga wasu jerin ansoshi, sai ki nuna ansan ki ta wurin wannan alama (✓) ko a zana a kan ansan da ya dace da ra’ayin ki. (Lura, za ki cire ansa daya ne tak daga jerin ansoshin).
- Ana roko da a yi gaskiya cikin ansan tambayoyin

**Sashe na A:** Sani game da mai cikawa

1. Shekaru ……………….........
2. Cikin ki sati nawa ne?..........
3. Aihuwan da ki ka yi kamin wanan, kin aife da daya ne ko tagwaye ko fiye ne?
4. Da daya
5. Yara biyu
6. Yara uku
7. Wanne makaranta mafi girma kin yi?
8. Ba makarantan boko
9. Firamari
10. Sakandari
11. Makaranta gaba da sakandari
12. Kasancewa da Aure?
13. Ba aure
14. Da aure
15. ’Yaya nawa ki ke da su? .......................
16. Menene aikin ki?.......................................
17. Menene a takaice abin biyan bukata da ke shigowa cikin gida a wata?
18. Kasa da N20,000
19. N20, 000 zuwa N40,000
20. N41,000 zuwa N 80,000
21. N81,000 zuwa N120,000
22. Sama da N120, 000
23. Wanne a cikin jerin ansoshin nan ke bayana dakyau iya biyan bukatan ki?
24. Talauci
25. Kasa da dai-dai dai-dai
26. Dai-dai dai-dai
27. Sama da dai-dai dai-dai
28. Arziki

**Sashe na B**: Gudanas da shayaswa da nono da kuma abubuwan da sun shafe yin haka.

1. Yin tunani kan haihuwa, wanne irin haihuwa ki ka yi?
2. Irin da aka saba bisa ga tsarin mutumtaka
3. Ta wurin na’uran forceps – wato abin da zai rike yaron ya fitar
4. Ta wurin cire yaro da inji
5. Ta wurin yanka
6. A lokacin nakuda, wanne irin maganin kashe zafi kika yi amfani da shi, in akwai?
7. Aluran kashin baya
8. Pethidine
9. Iskan shekawa
10. Maganin sa barci.
11. Ruwa
12. Babu kome ko kadan
13. Wasu (sai a fada wanne) .....................................................................
14. Menene nauyin yaron ki a lokacin haihuwa. Sai a fada haka bisa ga ma’aunin kilograms…………………………………………………………………………
15. Kafin haihuwan yaron ki na karshe, wanne shiri ki ka yi don ciyar da yaron a watani shidda na farko?
16. Madaran mama (nono)
17. Madaran yara da aka harhada
18. Madaran mama da kuma madaran yara da aka harhada
19. Ban yi wata shiri ba
20. Me ya sa ki ka yi tunanin ciyar da yaron ki ta wannan hanya? Sai a rubuta duka dalilan ……

……………………………………………………………………………………………………

………………………………………………………………………………………………………

……………………………………………………………………………………………………

………………………………………………………………………………………………………

1. A wanne lokaci ne kin fara ciyar da yaron ki bayan haihuwa?
2. Kasa da minti 30
3. Tsakanin minti 30 da sa’a 1
4. Tsakanin sa’a 1 da sa’o’i 4
5. Fiye da sa’o’i hudu bayan haihuwa
6. Wanne irin abinci ne yaron ki ya ci a farkon ciyaswa?
7. Madaran mama (nono)
8. Madaran yara da aka harhada
9. Wasu (sai a fada wanne)…………………………………………………………………………
10. Menene girman yaron ki ko kuma a wanne shekara ne kika fara gabatar wa yaron wani abinci banda madaran mama (nono) ........................
11. Menene babban dalilin da ya sa ki ka zabi wannan hanyan ciyas the yaron? …….........................

……………………………………………………………………………………………………

………………………………………………………………………………………………………

1. Idan kin ciyas da yaron ki da ruwa ko madaran da aka harhada ban da madaran mama a kwanakin farko na shayaswa da nono, kin yi haka ne don an baki shawara ne ko kuma don kin dai so yaron ki ya yi haka ne?
2. An bani shawara in bada koma menene
3. Na so ne in ba wa yaro na wani abu dabam
4. Na ba wa yarona madaran mama (nono) ne kawai a kwanakin farko.
5. Ko akwai wasu matsaloli da shayasda da yaro ta wurin madaran mama (nono) a kwanakin farko? ...........

…... in haka ne, menene matsalolin ……………………………………………………………………

……………………………………………………………………………………………….

……………………………………………………………………………………………….

………………………………………………………………………………………………..

1. Wanne a cikin jerin nan ya bayana da kyau ciyaswan yaron ki a sati daya (1), sati shidda (6), sati goma sha biyu (12) da kuma sati ashirin da hudu (24). (Sai a nuna alaman nan (✓) a inda ya cancanta. Za’a nuna alaman nan a wuri daya ne kachal a layin kowane tambaya, kuma za’a kasance da alaman nan guda hudu ne duka).

|  | Sati 1 | Sati 6 | Sati 12 | Sati 24 |
| --- | --- | --- | --- | --- |
| Madaran mama kawai |  |  |  |  |
| Madaran yara da aka harhada |  |  |  |  |
| Madaran mama tare da madaran yara da aka harhada |  |  |  |  |

1. Menene dalilan ciyas da yaron ki ta hanyan nan?

Dalilai na sati daya .................................................................................................................

..................................................................................................................................................

Dalilai na sati shidda………………………………………………………………………………

...................................................................................................................................................

Dalilai na sati goma sha biyu……………………………………………………………….....

......................................................................................................................................................

Sati ashirin da hudu....................................................................................................................

1. Ko kin dinga zuwa asibiti don bincike da lura a lokacin da kike da cikin yaron ki na yaron baya kafin haihuwa? ..............
2. Ko wani ya tatauna da ke game da ciyas da yaron ki a lokacin da kike da ciki? .……………
3. Wanene ya tatauna da ke game da ciyas da yaronki?
4. Likita
5. Mai lura da mutane a asibiti
6. Wasu (sai a fada wanne) ………………………………………………
7. A lokacin cikin ki na yaron ki a baya, kin karba koyaswa game da amfanin ciyar da yaro da madaran mama (nono)? ............................................................
8. A ina ki ka sami wannan koyaswa? ................................................................
9. Ta yaya abokai da iyali suka ciyas da ’ya’yansu a lokacin da suke jarirai?
10. Da yawan su sun basu madaran yara da aka harhada
11. Da yawan su sun bada madaran mama (nono)
12. Kusan rabin su sun mora madaran yara da aka harhada sai kusan rabi suka ba da madaran mama (nono)
13. Ban sani ba
14. Ta wanne hanya aka ciyar da ke a lokacin da kike jaririya?
15. Madaran mama (nono)
16. Madaran yara da aka harhada
17. Madaran mama da madaran yara da ka harhada
18. Ban sani ba
19. Idan kina da yara can baya, ta yaya kika ciyas da su a watani shidda na farko?

|  | Madaran mama kawai | Madaran yara da aka harhada kawai | Madaran yara da aka harhada tare da madaran mama |
| --- | --- | --- | --- |
| Yaya a cikin yaran |  |  |  |
| Yaro na biyu |  |  |  |
| Yaro na uku |  |  |  |
| Yaro na hudu |  |  |  |

1. An haife yaron ki a asibiti ne ko a gida?
2. Asibiti
3. Gida
4. A ranan da ki ka bar asibiti ko kuma bayan sa’o’i 48 idan kin haihu a gida, wannene ya bayana da kyau yanda kika ciyar da yaron ki?
5. Madaran mama (nono)
6. Mdaran yara da aka harhada
7. Madaran mama tare da madaran yara da aka harhada
8. Matsatsen madaran mama (nono)
9. Yaro bai fara shayaswa ba.
10. Ban tuna ba
11. Tun da kin haife yaron ki, ko kin ta samun wadannan ta dalilin shayas da yaron ta wurin madaran mama (nono)
12. Kumburan nono
13. Wasu irin fitarwa da kaikayi
14. Zafi a bakin nono
15. Babu ko daya daga cikin abubuwan da aka ambata a sama
16. Wasu, a fada wanne …………………….
17. Tun haihuwan yaron ki, wani ya taba ba ki shawara kada ki shayas da yaro da nono ko ki daina shayaswa da nono ko kuma ki sha magunguna da aka ayana maki?
18. I
19. A’a
20. Wanane ko kuma menene ya taimake ki don ki ci gaba da shayas da yaro da madaran mama (nono)?
21. Sanin kaina
22. Abokai da wasu uwaye
23. Maman maigidana
24. Wasu dangi
25. Masanan kimiya ta fannin lafiya (masu lura da mutane a asibiti da likitoci)
26. Tsara da kungiyoyin taimako
27. Kungiyoyin da sun tashi don kansu
28. Takardu/jaridu/talabijan
29. Wasu (sai a fada wanne)…………………………………………………
30. Wannene ko menene ya taimake ki kalila don cin gaban shayaswa da madaran mama (nono)?
    1. Sanin kaina
    2. Abokai da wasu uwaye
    3. Maman maigidana
    4. Wasu dangi
    5. Masanan kimiya ta fannin lafiya (masu lura da mutane a asibiti da likitoci)
    6. Tsara da kungiyoyin taimako
    7. Kungiyoyin da sun tashi don kansu
    8. Takardu/jaridu/talabijan
    9. Wasu (sai a fada wanne)…………………………………………………
31. Wanene ko menene ya shafe ki don ki daina shayaswa da madaran mama (nono)?
    1. Sanin kaina
    2. Abokai da wasu uwaye
    3. Maman maigidana
    4. Wasu dangi
    5. Masanan kimiya ta fannin lafiya (masu lura da mutane a asibiti da likitoci)
    6. Tsara da kungiyoyin taimako
    7. Kungiyoyin da sun tashi don kansu
    8. Takardu/jaridu/talabijan
    9. Wasu (sai a fada wanne)…………………………………………………
32. Ko kun hadu fata da fata da yaron ki bayan an haife shi/ta (a sa’a na farko bayan haihuwan)?
33. I
34. A’a
35. Ban tuna ba
36. Idan i ne, wanene ya taimake ki?
37. Ba a nuna mini ba
38. Ma’ aikatan jinya
39. Dalibai na fanin masu lura da mutane a asibiti da likitoci
40. Abokai da dangi
41. Likita
42. Masu hidima a dakunan asibiti
43. Wasu (a fada wanne) ……………………………………………………………………………
44. Sun kasance da ke a lokacin da kike shayaswa da madaran mama (nono)?
45. Ba a nuna mini ba
46. An tsaya dukan lokaci har sai da yaron ya yi baarci
47. An tafi lokacin da ake shayas da yaron amma an dawo don a dube ki
48. An tafi lokacin da ake shayas da yaron amma ba a dawo a dube ki ba
49. An tafi kafin yaron ya fara ci
50. Ta yaya kin same taimakon nan da amfani?
51. Ba a bani wani taimako ba a wannan lokaci
52. Da amfani matuka
53. Da amfani sosai
54. Ba amfani sosai
55. Babu amfani gaba daya
56. Kin same matsaloli da shayas da yaronki da nono a kwanakin farko?
57. I
58. A’a

Idan i, menene su…………………………………………………………………….

……………………………………………………………………………………………

………………………………………………………………………………………………

………………………………………………………………………………………………

1. Ko wani ya baki taimako bisa ga matsalar (matsala da shayaswa da nono) a kwanakin farko?
2. Ban samu wata matsala ba
3. Mai lura da mutane a asibiti ta/ya taimake ni
4. Likita ya/ta taimake ni
5. Aboki/dangi ya/ta taimake ni
6. Membobin kungiyan taimakon juna sun taimake ni
7. Wasu (a fada wanne)………………………………………………………
8. Bayan kin bar asibiti, ko kin sami wata ziyara daga daya daga cikin wadannan?
9. Ma’aikatan jinya
10. Likita
11. Babu ziyara a makoni biyu na farko
12. Wasu (a fada wanne)……………………………………………………………………….

Idan kin sami ziyara, guda nawa kika samu …………………………………..

1. Ko an ba ki bayani game da wadannan don taimako game da shayaswa da nono bayan kin tafi gida?
2. An bani bayani game da samun taimako
3. Kungiyan taimakon juna na gari (al’umma)
4. Wasu (a fada wanne) …………………………………………………………………………………
5. Ta yaya ya kasance maki da sauki ki sami taimako game da shayaswa da nono?
6. Ban nemi wani taimako ba
7. Da sauki
8. Da wuya
9. Ban iya samun taimako ba
10. Ta yaya kike shirin ciyar da jaririn da kike da cikin sa?
11. Madaran mama (nono)
12. Madaran yara da aka harhada
13. Madaran mama tare da madaran yara da aka harhada
14. Menene dalilin da zaki ciyar da yaron ki ta wannan hanya? .........................................................................

………………………………………………………………………………………………

……………………………………………………………………………………………………

………………………………………………………………………………………………………………………………………….........................................................................................................

1. Zuwa wanne tsawon lokaci kike shirin ciyar da yaron da madaran mama (nono) kawai …………
2. Don menene…………………………………………………………………………………………….....

………………………………………………………………………………………………………

1. A wanne shekara zaki daina ciyar da yaronki da madaran mama (nono)? …………………………
2. Idan kin san amfanin ciyar da yaro da madaran mama (nono), sai ki rubuta a nan …………

………………………………………………………………………………………………………

………………………………………………………………………………………………………

……………………………………………………………………………………………………

1. Idan kin san amfanin ciyar da yaro da madaran yara da aka harhada, sai ki rubuta a nan

……………………………………………………………………………………………………

…………………………………………………………………………………………………………………………………………………….........................................................................................

1. Ko kin taba ganin talle a talabijan, rediyo ko a jarida ko a ko’ina game da shayaswa da madaran mama (nono)? ........................................................................................................................................................
2. Ko kin taba ganin talle a talabijan, rediyo ko a jarida ko a ko’ina game da madaran yara da aka harhada? ………………………………………………………………………………………………………………………………………………….........................................................................................................................
3. Yin tunani akan bayani mafi taimako da kin samu game da shayaswa da mdaran mama (nono) tun haihuwan yaronki, wanene ko menene ya shafe ki sosai?
4. Sanin kaina
5. Abokai da wasu uwaye
6. Maigida
7. Mama na
8. Maman maigidana
9. ’Yan’uwa mata
10. Wasu dangi
11. Masanan kiwon lafiya
12. Takardu da jaridu
13. Wasu (a fada wanne) ……………………………………………………………………………
14. Tun haihuwan yaronki, kin taba shayas da shi/ita a wurin da ke kasance da mutane?
15. A’a – bana shayas a wurin da ke kasance da mutane
16. I – na shayas da yaro a wurin da ke kasance da mutane
17. I – na shayas da yaro da madaran yara da aka harhada a wurin da ke kasance da mutane
18. I – na shayas da yaro da matsatsen madaran nono
19. Kin taba samun matsala neman wurin da zaki shayas da yaro da nono a wurin da ke kasance da mutane? ....
20. Ko an taba tsayar da ke ko a sa ki rasa sakewa game da shayas da yaro a wurin da ke kasance da mutane? ...
21. Wanne a cikin wadannan ya bayana da kyau game da shayas da yaron ki da madaran mama (nono)?
22. Zan so da na shayas da yaro da nono na tsawon lokaci
23. Na shayas da yaro da nono a tsawon lokacin da na shirya
24. Na shayas da yaro da nono fiye da tsawon lokacin da na shirya
25. Menene tsawon lokacin da (a watani ko shekaru) ki ka shayas da yaronki na baya da madaran mama (nono)? .....................
26. Idan kin shirya kuma kin fara shayaswa da madaran mama (nono), wadanne dalilai ne suka sa kin daina? ..................................................................................................................

………………………………………………………………………………………………………

………………………………………………………………………………………………

……………………………………………………………………………………………

**Sashe na C: Game da Aiki**

1. Menene aikin mahaifin yaron? ................................................................
2. Ko kina aiki a lokacin da ki ka haife yaron ki na baya? ...................................................
3. Sa’o’i nawa ne kin yi ta aiki a rana lokacin da ki ka haife yaron ki na baya? ...........
4. Menene shakarun yaron ki a lokacin da ki ka koma aiki? ..............................................
5. Wadanne shiri (in akwai) ki ka yi don kula da yaron ki lokacin da ki na wurin aiki? ................................................................................................................................................

……………………………………………………………………………………………………....

……………………………………………………………………………………….........................

……………………………………………………………………………………............................

……………………………………………………………………………………………………………………………………………………........................................................................................

1. Ta yaya zaki bayana aikin ki kafin kin haife yaron ki?
2. Aiki don a biya ni ko in sami riba
3. Neman aiki na na farko
4. Ba ni da aiki
5. Dalibi
6. Lura da gida/iyali
7. Rashin iya yin aiki don daddaden cuta/nakasa
8. Wasu (a fada wanne) …………………………………………………
9. Kin yi (ko kina yi) aiki a kalkashin wani ko kin yi (ko kina yi) aiki na kanki?
10. Aiki kalkashin wani
11. Aikin kaina, da ma’aikata da nake biya a kalkashi na
12. Aikin kaina, babu ma’aikata da nake biya a kalkashina
13. Taimakon dangi/wasu (bana karban tsararen albashi)
14. Lura da gida/iyali
15. Kina shirin fara aiki a cikin shekaru biyu masu zuwa?
16. I – aikin cikakken lokaci
17. I – aikin dauke yan sa’o’i kawai
18. A’a
19. Ban sani ba
20. Ina aiki yanzu
21. Ko mai baki aiki ya tanada ma ki kayan aiki don matse nono ko kuma shayaswa da yaron ki da nono idan kina son yin haka?
22. I – in matsa nono
23. I – in shayas da yaro da nono
24. A’a – babu ko daya
25. Bai shafe ni ba
